# Postoperative nursing after tracheoplasty for congenital tracheal stenosis

**DOI:** 10.3389/fped.2026.1745567

**Published:** 2026-05-19

**Authors:** Ying Qiu, Shujuan Sun, Pengrong Huang, Wen Sun, Xia Sun

**Affiliations:** 1 Department of Cardiac Surgery, children’s Hospital of Shandong University (Jinan children’s Hospital), Jinan, China; 2 Department of Geriatrics, Shandong Provincial Hospital Affiliated to the First Medical University, Jinan, China

**Keywords:** congenital tracheal stenosis, nursing, pediatric, postoperative complications, tracheoplasty

## Abstract

**Background:**

To explore targeted nursing strategies for managing postoperative complications in children with congenital tracheal stenosis (CTS) following tracheoplasty.

**Methods:**

A total of 263 children with CTS who underwent tracheoplasty were retrospectively analyzed. Key aspects of postoperative nursing included body positioning, endotracheal intubation management, invasive mechanical ventilation management, airway care, and the identification and management of common postoperative complications.

**Results:**

Twelve children died during hospitalization, yielding an overall mortality rate of 4.5%. Granulation tissue hyperplasia was observed in 18 cases and was treated with endoscopic laser cauterization and/or forceps removal. Anastomotic scar contracture occurred in 34 cases and was treated with endoscopic balloon dilatation. Severe tracheomalacia affecting ventilation was identified in 43 patients, among whom 9 required tracheal silicone stent implantation. A total of 251 surviving children were followed up for 1–56 months (median: 13 months). Two children with preoperative bronchiolitis obliterans remained dependent on intermittent oxygen therapy at the final follow-up. Respiratory symptoms were significantly improved in the remaining survivors.

**Conclusion:**

Meticulous postoperative airway nursing care in children with CTS can improve surgical outcomes, reduce complications, and facilitate postoperative recovery.

## 1 Introduction

Congenital tracheal malformations are rare but potentially life-threatening conditions, with an estimated incidence of approximately 1 in 64,500 live births (1, 2). In paediatric populations, they most commonly present with stridor and respiratory distress (3). Congenital tracheal stenosis (CTS) is typically characterized by the absence of the posterior membranous portion of the trachea, which is replaced by complete tracheal rings (4, 5). In recent years, advances in surgical techniques have markedly improved the prognosis of children with CTS, and tracheoplasty has become the treatment of choice (6–8).

However, surgical intervention introduces additional challenges for postoperative management. Factors such as tracheal shortening, intraoperative mucosa injury, cardiopulmonary bypass–related effects, and the simultaneous correction of associated cardiovascular anomalies necessitate close postoperative monitoring and specialized nursing care. Effective nursing management, including early identification and timely intervention for complications, is therefore critical to reducing postoperative morbidity and mortality.

Compared with Western countries, where pediatric tracheoplasty has been established since the late 1980s to early 1990s, its adoption in China occurred relatively later, with the first reports of surgical outcomes emerging around 2009 (9–11). Currently, only a limited number of specialized centers perform this procedure, and the number of reported cases remains relatively small. At present, standardized protocols for postoperative nursing and monitoring has not been fully established. Based on our institutional experience in the surgical treatment and postoperative care of CTS in recent years, we summarize key nursing strategies to provide practical guidance for clinical practice.

## 2 Methods

### 2.1 General information

A total of 263 pediatric patients with CTS who underwent surgical treatment in our institution between February 2017 and December 2023 were enrolled in this study. The diagnosis was confirmed using electrocardiography, echocardiography, and spiral computed tomography (CT) in all patients. The cohort comprised 158 males and 105 females. The age at surgery ranged from 14 days to 10 years, with a mean average of 13.5 months. Body weight at the time of operation ranged from 2.5–39.0 kg (median: 10.7 kg). With respect to tracheal stenosis characteristics, 239 cases had long-segment stenosis (involving > 30% of the total trachea length), while 24 had short-segment stenosis (<30%). Absence of tracheal cartilage rings was observed in 6 cases. Associated airway anomalies included bridging bronchus (*n* = 20), tracheal bronchus (*n* = 9), tracheal diverticulum (*n* = 12), and pulmonary dysplasia (*n* = 15). Cardiovascular comorbidities were common, including pulmonary artery sling (*n* = 153), patent ductus arteriosus (*n* = 40), atrial septal defect (*n* = 61), ventricular septal defect (*n* = 40), pulmonary artery stenosis (*n* = 4), pulmonary atresia (*n* = 3), coarctation of the aorta (*n* = 3), and double aortic arch (*n* = 3). Vocal cord paralysis was present in 12 cases, and 18 cases were born prematurely. Some patients had multiple (2–4) concomitant congenital cardiovascular anomalies. A total of 53 patients had a history of prior cardiovascular surgery, including repair of tetralogy of Fallot, pulmonary artery sling correction, ventricular septal defect repair, atrial septal defect repair, patent ductus arteriosus ligation or occlusion, and main pulmonary artery window repair. One patient had spontaneous closure of a ventricular septal defect. Preoperative airway management included endotracheal intubation in 157 patients and a history of tracheotomy in 3 patients. Early repeated balloon dilatation was performed in 4 cases. In addition, 11 patients underwent early tracheal metallic stent implantation. among them, 1 case had a stent in place for 4 days, while the remaining 10 patients had implanted duration ranging from 21.6–74.2 months (median: 56.8 months) (Table 1).

**Table 1:** Baseline characteristics of patients undergoing tracheal or bronchial reconstruction (*n* = 263).

**Baseline characteristics**	**Number**
The average age at surgery (IQR: Q1–Q3)	13.5 months (14 days to 10 years)
Weight during surgery (IQR: Q1–Q3)	10.7 kg (2.5–39 kg)
Preoperative intubation, *n* (%)	157 (59.6)
Preoperative tracheostomy, *n* (%)	3 (1.1)
Insertion of metallic tracheal stent *n* (%)	11 (4.1)
Tracheal or bronchial lesions, *n* (%)	
Carina bronchus	20 (7.6)
Tracheobronchial	9 (3.4)
Absence of cartilage rings	6 (12.1)
Tracheal diverticulum	12 (4.5)
Preoperative comorbidities, *n* (%)	
Congenital pulmonary artery sling	153 (58.1)
Patent ductus arteriosus	40 (15.2)
Atrial septal defect	61 (23.1)
Prematurity	18 (6.8)
Ventricular septal defect	40 (15.2)
Pulmonary atresia	3 (1.1)
Pulmonary hypoplasia	15 (5.7)
Pulmonary stenosis	4 (1.5)
Laryngeal paralysis	12 (4.5)
Coarctation of the aorta	3 (1.1)
Double aortic arch	3 (1.1)

### 2.2 Treatment

#### 2.2.1 Preoperative preparation

All children underwent preoperative tracheoscopy, budesonide inhalation was administered three times consecutively at 20 min intervals. A total of 39 children received preoperative endotracheal intubation and mechanical ventilation, including 32 who were transferred from other institutions with an indwelling endotracheal tube. In addition, nine children were referred after failed weaning from mechanical ventilation following thoracotomy at other hospitals; among them, 8 had undergone surgical correction of congenital cardiovascular malformations, and one had undergone pulmonary artery sling repair combined with slide tracheoplasty. Mechanical ventilation was delivered using Pressure Regulated Volume Control (PRVC) mode, with a tidal volume of 8–10 mL/kg and age-adjusted respiratory rates to ensure adequate minute ventilation. Surgery was performed after normalization of leukocyte count and body temperature in intubated patients.

#### 2.2.2 Surgical approach

All patients underwent surgical correction of tracheal stenosis under general anesthesia with cardiopulmonary bypass (CPB), with concomitant anomalies addressed during the same procedure. Through a median sternotomy, the sternum was longitudinally divided, the pericardium was opened and suspended, and CPB was established via cannulation of the aorta, superior vena cava, and inferior vena cava following systemic heparinization. Among them, 36 children with intracardiac malformations underwent surgery under hypothermic circulatory arrest with simultaneous tracheoplasty. In 88 children with cardiovascular malformations, cardiac defects were corrected under normothermic CPB, followed by tracheoplasty. The remaining patients underwent tracheoplasty under normothermic CPB. In terms of surgical techniques, slide tracheoplasty was performed in 261 cases, resection with end-to-end anastomosis in 1 case, and carinal reconstruction in 1 case. Metallic stents were simultaneously removed in 11 patients (Table 2).

**Table 2:** Treatment methods for children with congenital bronchial stenosis after surgery (*n* = 263).

Treatment methods	Numerical value (%)
Preoperative bronchoscopy	263 (100)
Endotracheal intubation ventilation	18 (6.8)
Surgical correction under hypothermia cardiac arrest	36 (13.6)
Ambient temperature surgical correction	88 (33.4)
Slide tracheal reconstruction	261 (99.2)
Segmental resection and end-to-end anastomosis	1 (0.3)
Reconstruction of the carina	1 (0.3)
Removal of metal stent during surgery	11 (4.1)

## 3 Results

### 3.1 Surgical outcomes

The duration of cardiopulmonary bypass ranged from 46 to 388 min, with a median of 150 min. The length of tracheal stenosis ranged from 0.5∼7.8 cm, with a median of 4.0 cm. Among the 263 cases, 11 died during hospitalization and 1 died suddenly 6 months after discharge, yielding an overall mortality rate of 4.5%. A total of 251 patients survived the operation. The Follow-up period ranged from 1 to 56 months, with a median of 13 months. Two patients had preoperative bronchiolitis obliterans and remained mildly activity-limited at the end of follow-up, still requiring home oxygen therapy. Follow-up tracheoscopy demonstrated satisfactory tracheal recovery. In the remaining patients, respiratory symptoms were significantly improved, with no significant differences compared with age-matched peers (Table 3).

**Table 3:** Postoperative surgical results of children with congenital bronchial stenosis.

Surgical results	Numerical value
Duration of extracorporeal circulation in patients	46–388 min
Death	12 (total)
Unable to we from the ventilator postoperatively	4 (33.3%)
Complicated cardiovascular malformation	2 (16.6%)
Ileus with colostomy	1 (8.3%)
Right lung hypoplasia	1 (8.3%)
Left main bronchus softening	3 (25%)
Unknown cause	1 (8.3%)

### 3.2 The main cause of death in children

In the early postoperative period, 4 cases had unsatisfactory outcomes following tracheoplasty and could not be weaned from mechanical ventilation. Among them, 2 cases had complex cardiovascular malformations and experienced prolonged cardiopulmonary bypass, resulting in severe low cardiac output, anastomotic dehiscence, and pulmonary hemorrhage, and could not be weaned from CPB. One child with right lung hypoplasia developed compression of the left pulmonary artery after tracheal reconstruction and could not be weaned from CPB. Another child, who had previously underwent colostomy for intestinal obstruction, died of sepsis due to recurrent obstruction and ineffective treatment. In addition, 3 cases were complicated with left main bronchomalacia and stenosis of the right main bronchus orifice, resulting in difficulty in postoperative ventilator weaning. One patient died suddenly 6 months after discharge due to unknown causes.

### 3.3 Postoperative complications and treatment

Among the 216 children who were successfully discharged, 148, 29, and 13 were extubated at 24, 48, and 72 h postoperatively, respectively. The remaining 26 children were extubated between 74.5 and 438.8 h due to pulmonary infection, tracheomalacia, and other complications. One case developed anastomotic leakage above the carina; however, no respiratory symptoms were observed, and the lesion resolved spontaneously within 1 month without specific treatment.

Anastomotic granulation tissue hyperplasia occurred in 18 cases and was treated with endoscopic laser cauterization and/or forceps resection, with favorable outcomes. Among 34 cases with anastomotic scar contracture, 33 showed improvement after endoscopic balloon dilatation, while 1 had a suboptimal response. A silicone stent was placed in this patient and removed after 5 months, with satisfactory airway patency.

Severe airway malacia affecting ventilation was observed in 43 patients. Of these, 33 improved with continuous positive airway pressure (CPAP) support. Nine patients required silicone stent placemen, which was removed after 1–3 months, with good recovery. One patient had persistent moderate malacia with limited activity and remained under follow-up (Table 4).

**Table 4:** Postoperative complications and management (*n* = 263).

Postoperative complications	Percentage (%)
Pulmonary infection, tracheal softening	26 (9.8)
Anastomotic granulation hyperplasia	18 (6.8)
Anastom scar contracture	34 (12.9)
Severe tracheal softening affecting ventilation	43 (16.3)
Tracheal stent placement 9 Anastom gap	2 (0.7)

## 4 Discussion on postoperative observation and nursing

The marked heterogeneity and complexity of the CTS patient population pose significant challenges for clinical management. The standard of care for CTS has evolved toward an integrated, multidisciplinary team-based approach (12, 13), multidisciplinary, team-based approach. This model has been associated with improved perioperative outcomes, including reduced duration of mechanical ventilation and shorter ICU and hospital stay (14).

### 4.1 Postural management

Postural positioning plays a critical role in postoperative airway healing in children and is an important determinant of surgery success (15). Upon admission to the ICU, nursing staff were fully informed of the severity of tracheal stenosis and the surgical approach, and patient positioning was managed according to individualized requirements. In children with short-segment stenosis, whether treated with segment resection and end-to-end anastomosis or slide tracheoplasty, anastomotic tension is generally low, and no specific postoperative positioning is required. In contrast, children with long-segment tracheal stenosis tend to have higher anastomotic tension. Previous studies have suggested maintaining the head in a mildly flexed position with pillow support after surgery, without the need for chin-to-chest fixation (12, 16). Infants are more susceptible to airway compression of the lateral lung segment in the supine position due to the increased compliance of the chest wall (17). In our practice, patients were positioned supine with appropriate pillow support or with slight head flexion postoperatively, while excessive neck extension was strictly avoided during holding, sitting, or standing. For children capable of independent sitting or ambulation, additional attention was given to nursing care and caregiver education to ensure appropriate positioning.

### 4.2 Nursing of endotracheal intubation

Tracheoplasty inevitably results in shortening of the trachea, which is more pronounced in children with long-segment tracheal stenosis. Intraoperatively, the tip of the endotracheal tube is routinely positioned approximately 1 cm above the carina to ensure adequate airway support while minimizing the risk of injury. Excessive insertion depth may lead to carinal damage and severe complications. Therefore, precise postoperative management of endotracheal tube depth is essential, with the tip ideally maintained about 1 cm proximal to the carina. In typical cases, tracheal length may be reduced by approximately 1–3 cm, with corresponding cephalad displacement of the carina. Consequently, the appropriate tube depth should be reassessed based on postoperative anatomical changes rather than preoperative measurements. Although tube positioning is initially confirmed intraoperatively under bronchoscopy, subsequent changes in patient position and loosening of fixation may alter tube depth.

Therefore, careful postoperative monitoring is required. Tube position should be regularly assessed and documented using external reference points (e.g., distance to the alae nasi or incisors), and verified when necessary by chest radiography or bronchoscopy. In this cohort, routine bedside chest radiography after ICU admission identified 4 cases in which the tube tip was positioned too close to the carina, all of which were promptly corrected.

### 4.3 Management of invasive ventilator

All patients were managed with PRVC ventilation postoperatively. A significant increase in peak airway pressure warrants prompt evaluation. After excluding ventilator-related factors, the following causes should be considered: First, airway obstruction due to retained secretions or blood clot may lead to elevated airway pressure, which typically decreases after suctioning. If airway pressure does not improve significantly and secretions are minimal, fiberoptic bronchoscopy is recommended to assess airway patency and guide further management. Second, pulmonary edema or atelectasis should be considered, particularly in patients with prolonged cardiopulmonary bypass or severe preoperative pulmonary infection. In such cases, the airway may appear patent on bronchoscopy, while chest radiography may reveal pulmonary exudation or collapse. Pulmonary edema requires optimization of fluid management, including diuresis and adjustment of positive end-expiratory pressure (PEEP), whereas atelectasis may be managed with positioning, chest physiotherapy, and, if necessary, bronchoscopic intervention. Third, residual stenosis or airway distortion following tracheoplasty should be suspected. This can be confirmed by fiberoptic bronchoscopy and may require further surgical or interventional treatment.

Endotracheal intubation following tracheoplasty can function as temporary “scaffold”, to support the reconstructed airway. Previous studies have suggested that extubation is generally considered 3–7 days after surgery (18). However, respiratory complications remain common after slide tracheoplasty and are often commensurate with underlying cardiac or pulmonary comorbidities. More recent evidence indicates that early extubation is feasible in over 50% of cases (19). In our experience, once tracheal stenosis has been adequately corrected, prolonged intubation may be detrimental, as it impairs mucociliary clearance, compromises the airway barrier, and increases the risk of infection. Therefore, a strategy of early extubation was adopted. Postoperatively, sedation and neuromuscular blockade were routinely administered, and sedation was discontinued on the first postoperative day to allow clinical assessment. When hemodynamics stability and satisfactory arterial blood gas results were achieved, extubation was performed as soon as possible.

### 4.4 Respiratory tract management

Airway infections is an important risk factor for the development of tracheal stenosis (18); therefore, meticulous postoperative airway management is essential.

First, bloody airway secretions are common after tracheoplasty. Due to postoperative sedation, children are often unable to clear secretions effectively, necessitating timely suctioning. In most cases, secretions gradually change from fresh blood to old blood–tinged sputum. Persistent fresh blood beyond the early postoperative period may indicate minor bleeding from suture sites or mucosal injury. In such cases, topical epinephrine (1:10,000, 0.5–1.0 mL) can be administered via the endotracheal tube, which is generally effective after repeated applications. However, ongoing fresh bleeding should raise concern for active hemorrhage and warrants prompt evaluation with fiberoptic bronchoscopy. It should be noted that excessive use of epinephrine may induce local vasoconstriction, potentially leading to mucosal ischemia or necrosis. In our cohort, one case of tracheal wall discoloration was considered to be associated with high-dose epinephrine exposure. Therefore, we recommend limiting epinephrine use to the early postoperative period and avoiding repeated administration. Second, the volume and characteristics of sputum provide important clinical clues for fluid management. Large volumes of thin secretions may indicate fluid overload and should prompt fluid restriction and diuresis stimulation targeting a negative fluid balance. In contrast, thick, tenacious sputum suggests inadequate hydration and requires volume repletion, reduced diuretic use, and airway humidification, such as saline nebulization.

Third, aerosol inhalation. It includes oxygen-driven nebulization of inhaled budesonide and ultrasonic nebulization of normal saline. During tracheoplasty, trachea opening and periods of respiratory arrest may lead to alveolar collapse, while cardiopulmonary bypass–related injury can contribute to pulmonary edema and bronchospasm. Therefore, postoperative inhalation of corticosteroids may help reduce airway edema and relieve bronchospasm. In addition, previous studies have reported that inhaled budesonide can prevent and treat granulation tissue hyperplasia at the a anastomotic site (20). It should be noted that high-dose or prolonged use corticosteroids may impair anastomotic healing and increase the risk of local infection. We recommend that budesonide aerosol inhalation be routinely administered for 5–7 days postoperatively, generally not exceeding 2 weeks. Nebulized normal saline (10 mL per session) is primarily used to dilute airway secretions and prevent mucosal dryness, and can be repeated every 2–6 h according to sputum viscosity.

Fourth, education and caregiver guidance after transfer from the intensive care unit. Following transfer from the intensive care unit, children may continue to produce excessive sputum due to irregular tracheal mucosa and impaired mucociliary clearance after tracheoplasty. As the mucosal epithelium gradually repairs over time, this process should be clearly explained to patients and their caregivers to alleviate anxiety. In addition, caregivers should be encouraged to perform chest physiotherapy regularly. Medical staff should provide instruction and demonstrate on appropriate postural percussion techniques in different positions to ensure effective airway clearance.

### 4.5 Identification and nursing of major postoperative complications

(1) Anastomotic dehiscence. Anastomotic dehiscence is a serious complication after tracheoplasty, often secondary to mediastinal infection or excessive anastomotic tension. In cases of a small defect, clinical manifestations may include subcutaneous emphysema, mediastinal emphysema, pneumothorax, and minor air leakage from the drainage system. As small defects may heal spontaneously, close observation and documentation of air leakage are generally sufficient. In contrast, large defects may be associated with decreased oxygen saturation and rapid hemodynamics deterioration, and severe cases may require thoracotomy or reoperation (13, 16). Therefore, careful monitoring is essential. Nursing staff should regularly assess skin changes, auscultate bilateral breath sounds, monitor the drainage system, and closely observe vital signs. In this cohort, anastomotic dehiscence was detected in two patients during routine bronchoscopy 14 days postoperatively. Enhanced monitoring was implemented, including frequent assessment of vital signs and oxygen saturation, regular examination of chest and abdominal skin (every 1–2 h), auscultation of bilateral lung sounds, and strict adherence to aseptic technique during airway suctioning. (2) Tracheomalacia. Tracheomalacia is a common complication following tracheoplasty, with an incidence of approximately 20%–30% (21). Clinically, patients may remain stable during endotracheal intubation but develop dyspnea, labored breathing, and poor response to aerosol therapy and airway suctioning after extubation. The diagnosis can be confirmed by bronchoscopy. Noninvasive ventilation with continuous positive airway pressure (PACP) is commonly used for management. In most patients, respiratory symptoms improve with ventilatory support, which is typically maintained for 1–2 weeks depending on clinical condition. Nursing care should focus on preventing complications such as pressure injury, atelectasis, and pulmonary infection. In some cases, reintubation and mechanical ventilation may be required. Although tracheomalacia often improves over time, a subset of patients with severe symptoms may require endotracheal stent placement. Following stent implantation, airway secretion clearance may be impaired; therefore, airway management measures, including aerosol therapy, chest physiotherapy, and suctioning, should be intensified.

## 5 Conclusions

CTS is a rare but potentially life-threatening airway disorder, frequently associated with complex congenital heart disease, a making surgical treatment and perioperative care highly challenging. Our institution is a specialized center for the management of CTS in China, with extensive experience in treating such patients. A multidisciplinary team approach, along with standardized protocols for diagnosis, treatment, and nursing care, has been established. In this study, we summarized the postoperative nursing experience in 263 children with CTS, including general care, postural management, endotracheal tube management, invasive ventilator support, airway management, and the identification and management of postoperative complications (Table 5). These findings may provide a useful reference for clinical nursing practice and contribute to improving surgical outcomes in children with CTS.

**Table 5:** Summary of nursing standards, potential complications, and their prevention and detection methods.

Nursing Category	Nursing Standards	Possible Complications	Prevention & Detection
Postural Care	Short-segment stenosis: no strict position limit. Long-segment stenosis: supine with slight head-forward tilt; avoid excessive head extension; strengthen health education.	Anastomotic dehiscence	Observe subcutaneous emphysema, air leaks, SpO_2_, hemodynamics; bedside chest x-ray if needed.
Intubation Care	Tube tip 1 cm above carina.	Carina injury; accidental extubation	Record tube depth; check fixation; confirm position by chest x-ray/bronchoscopy.
Ventilator Management	Monitor peak inspiratory pressure (PIP).	Airway obstruction by scab/clot	Pressure drops quickly after suction.
		Pulmonary edema, atelectasis	Diuresis/PEEP adjustment; chest physiotherapy; bronchoscopy if needed.
		Residual stenosis, airway distortion	Diagnosed by bronchoscopy.
Bloody Secretion Care	Suction timely; 1:10,000 epinephrine saline 0.5–1.0 mL for local hemostasis (3–5 times).	Mucosal ischemia/necrosis	Use within 24 h, max 8 times daily.
Sputum Management	Suction as needed.	Mucosal injury	Diuresis adjustment; saline nebulization for thick sputum.
Nebulization Care	Budesonide 5–7 d; saline 10 mL q2–6 h.	Delayed healing, local infection	Avoid high-dose/long-course steroids.

## Data Availability

The raw data supporting the conclusions of this article will be made available by the authors, without undue reservation.
